# Case Report: Pathologic Complete Response to Pembrolizumab in Combination With Neoadjuvant Chemotherapy in a Patient With Stage IIB Squamous Lung Cancer

**DOI:** 10.3389/fsurg.2020.601805

**Published:** 2020-11-27

**Authors:** Lin Zhang, Wuqian Mai, Wenyang Jiang, Qing Geng

**Affiliations:** ^1^Department of Thoracic Surgery, Renmin Hospital of Wuhan University, Wuhan, China; ^2^Department of Cardiology, Union Hospital, Tongji Medical College, Huazhong University of Science and Technology, Wuhan, China; ^3^Key Lab of Molecular Biological Targeted Therapies of the Ministry of Education, Union Hospital, Tongji Medical College, Huazhong University of Science and Technology, Wuhan, China

**Keywords:** PD-1/PD-L1, pembrolizumab, lung cancer, neoadjuvant therapy, pathological complete response (PCR)

## Abstract

The emergence of PD-1 antibodies has radically changed the therapeutic profiles of lung cancer, which has the highest incidence and mortality rate among all cancers worldwide. Pembrolizumab, an anti-PD-1 antibody, has been proven to have strong anti-tumor activity in a variety of clinical studies. Here, we described a patient with stage IIB squamous lung cancer who has benefited from a preoperative pembrolizumab plus chemotherapy regimen. The addition of pembrolizumab to neoadjuvant chemotherapy before surgery led to a pathologic complete response and the avoidance of left pneumonectomy. This case highlights the effect of neoadjuvant pembrolizumab plus chemotherapy on non-small cell lung cancer patients and has provided a promising choice in future clinical practice.

## Introduction

Lung cancer is the tumor with the highest incidence and mortality rate both in China and worldwide (1). Among all types of lung cancer, non-small cell lung cancer (NSCLC) accounts for 85% of the total cases (1). The optimal treatment for NSCLC used to be surgery when the tumor is resectable. However, many patients are in a relatively late stage when diagnosed, which is an important reason for the poor prognosis of lung cancer. In recent years, the advent of immune checkpoint inhibitors (ICIs) has dramatically changed the landscape of lung cancer therapy (2). Among all ICIs applied in clinical studies, anti-programmed cell death protein 1(PD-1)/programmed cell-death protein 1 ligand 1 (PD-L1) antibodies are the most successful ones (3). Pembrolizumab is an anti-PD-1 antibody approved by the US Food and Drug Administration for the treatment of non-small cell lung cancer (2). In previous studies, pembrolizumab as a first-line therapy has shown satisfying anti-tumor activity in advanced or metastatic NSCLC patients (4–6). In addition, the study investigating the effect of pembrolizumab plus chemoradiation as a neoadjuvant therapy in resectable NSCLC patients has been included in the American Society of Clinical Oncology meeting library (7). However, no prospective studies have been published to investigate the effect of pembrolizumab plus chemotherapy as a neoadjuvant therapy in NSCLC patients yet. Here, we presented a case with stage IIB NSCLC who had received neoadjuvant pembrolizumab plus chemotherapy before surgery, was assessed as having a complete pathologic response, and who avoided pneumonectomy. The following case was presented in accordance with the CARE reporting checklist.

## Case Description

**Figure 1:**
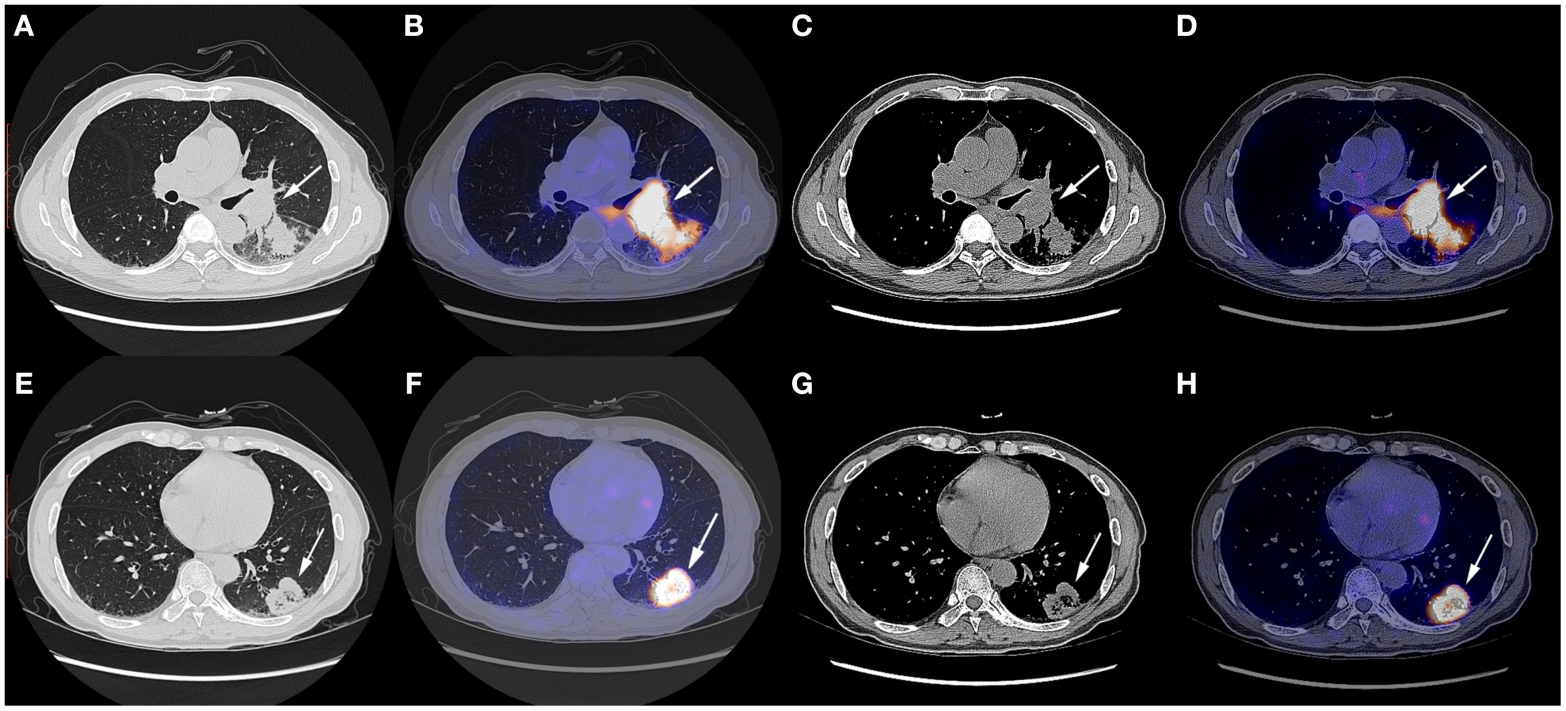
Chest CT scan and fused PET-CT images of the masses on October 9th, 2019. **(A–D)** The SUVmax of the mass adjacent to the hilum was 25.3 and the size of it was 3.6 cm × 5.2 cm × 4.8 cm; **(E–H)** The SUVmax of the mass on the basal segment was 12.5 and the size of it was 3.8 cm × 3.6 cm × 3.8 cm. White arrows indicate the locations of the masses.

A 57 year-old Chinese man with the complaint of a dry cough for more than 1 month was admitted to the Department of Thoracic Surgery. The patient had a smoking history of over 20 years, smoking two packs per day, and several years' history of hemorrhoids. He had not undergone any surgery before and had no family history of tumor. His physical status score was 0. The chest CT scan on October 9th, 2019 showed a soft tissue shadow on his left lower lobe and a left hilum space-occupying lesion (Figure 1). The masses were consistent on CT imaging, indicating that the one next to the left hilum was an enlarged hilum lymph node. The CT scan also showed an inflammatory lesion and obstructive lesion on the left lower lobe (Figure 1). PET-CT scan on October 9th, 2019 indicated a dorsal segment soft tissue mass on the left lower lobe adjacent to the hilum, with a size of 3.6 cm × 5.2 cm × 4.8 cm and an SUVmax of 25.3, and a basal segment soft tissue mass, with a size of 3.8 cm × 3.6 cm × 3.8 cm and an SUVmax of 12.5 (Figure 1); the masses had an increased metabolic rate and were considered to be malignant lesions. Multiple mediastinal and right hilum lymph nodes were detected with an increased metabolic rate; the lymph nodes were considered to be inflammatory lesions. Otherwise, no distant metastatic signs were exhibited on PET-CT. No abnormal signs were observed on brain MRI, either.

**Figure 2:**
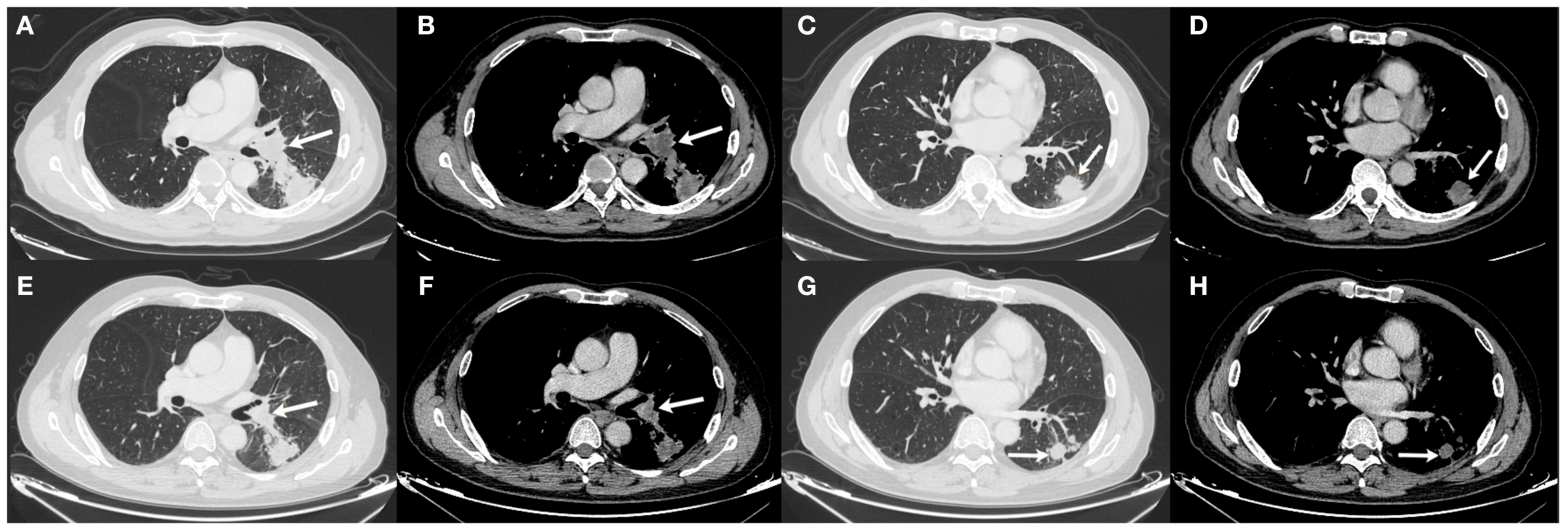
Chest CT scan of the masses on December 6th, 2019 **(A–D)** and March 15th, 2020 **(E–H)**. **(A,B)** The size of the mass adjacent to the hilum was 2.2 cm × 3.3 cm × 2.1 cm on December 6th, 2019; **(C,D)** The size of the mass on the basal segment was 2.9 cm × 3.0 cm × 2.1 cm on December 6th, 2019; **(E,F)** The size of the mass adjacent to the hilum was 2.1 cm × 2.5 cm × 1.9 cm on March 15th, 2020; **(G,H)** The size of the mass on the basal segment was 2.0 cm × 1.9 cm × 1.8 cm on March 15th, 2020. White arrows indicate the locations of the masses.

**Figure 3:**
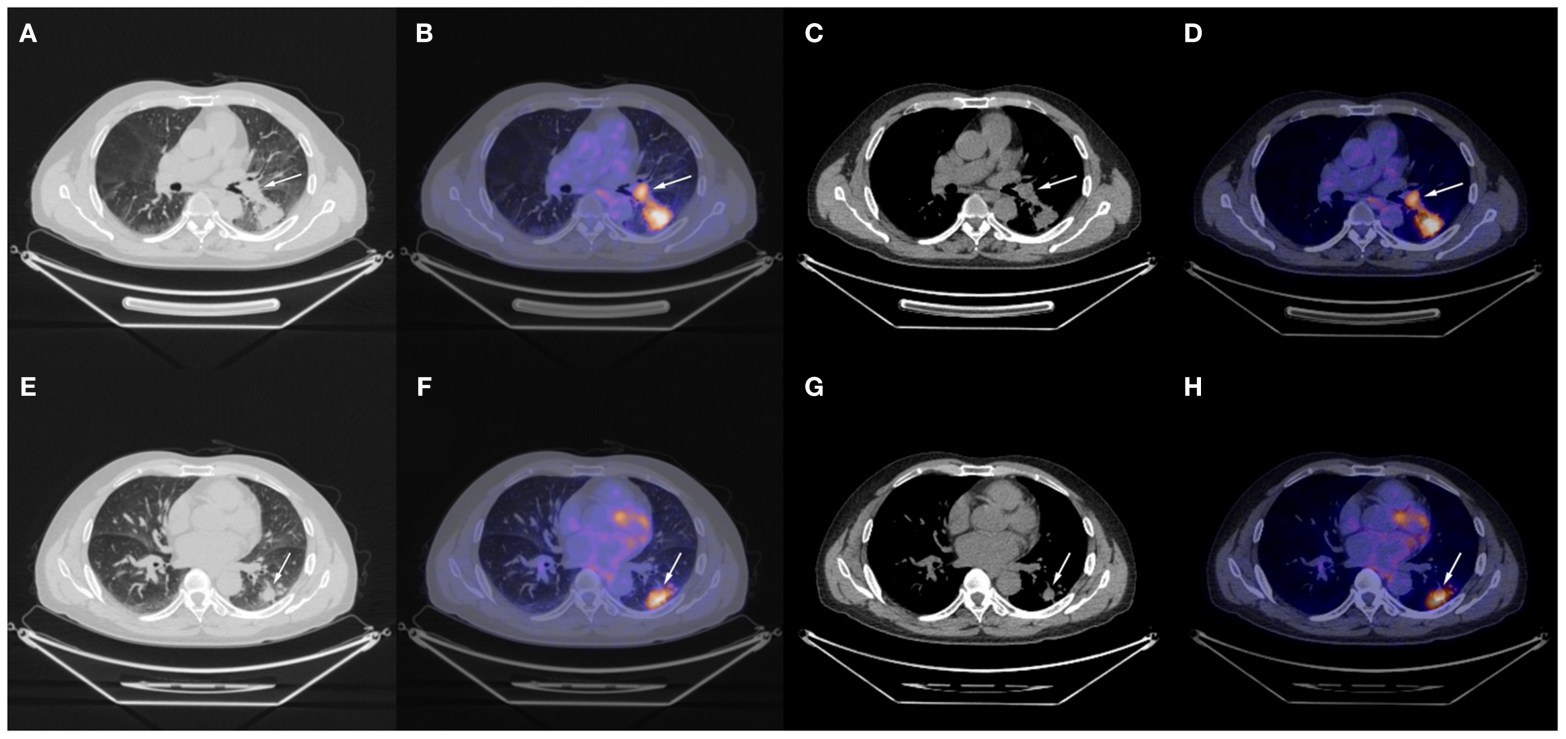
Chest CT scan and fused PET-CT images of the masses on April 28th, 2020. **(A–D)** The SUVmax of the mass adjacent to the hilum was 8.4 and the size of it was 1.7 cm × 2.3 cm × 2.0 cm; **(E–H)** The SUVmax of the mass on the basal segment was 8.4 and the size of it was 1.7 cm × 1.8 cm × 1.6 cm. White arrows indicate the locations of the masses.

The patient underwent needle biopsy on October 12th, 2019 and the pathologic result showed poorly differentiated squamous carcinoma. The patient refused to take any invasive examinations after needle biopsy, thus mediastinal staging was not performed. The specimen was sent for PD-L1 detection and tumor proportion score (TPS) was 80%, indicating a high expression of PD-L1. Thus, the clinical staging for this patient was cT2aN1M0, IIB according to the 8th edition lung cancer stage classification system (8). Because of the enlarged lymph node proximal to the hilum, the surgery plan was considered to be pneumonectomy. Thus, in order to reduce the size of the tumor and preserve residual lung function, this patient received neoadjuvant chemotherapy (gemcitabine 1.6 g, d1, d8 plus cisplatin, 60 mg, d1, d2, Q3W). The chemotherapy plan was implemented for five cycles from October 17th, 2019 to March 17th, 2020 and pembrolizumab (200 mg, d1, Q3W) was added from the second cycle. Due to the outbreak of COVID-19, the plan had to be suspended after the fourth cycle. The fifth cycle was initiated on March 17th, 2020. The CT scan on December 6th, 2019 after two cycles of neoadjuvant therapy and March 15th, 2020 after four cycles of neoadjuvant therapy showed shrinking masses on the left lower lobe (Figure 2). During the treatment, the adverse effects in this patient included vomiting, thrombocytopenia, and occasional chest distress. The platelet count of the patient was 61.00 × 10^9^/L on October 31st, 2019. All the symptoms were alleviated after symptomatic treatment. The PET-CT scan on April 28th, 2020 showed the mass adjacent to the hilum on the left lower lobe to be 1.7 cm × 2.3 cm × 2.0 cm, with the SUVmax being 8.4 (Figure 3). The mass on the basal segment was 1.7 cm × 1.8 cm × 1.6 cm, with an SUVmax of 8.4 (Figure 3). Finally, the patient underwent left lower lobectomy on April 30th, 2020 and the intraoperative pathologic report showed a benign lesion of the left lung nodules and no tumor in the resected lymph nodes. The postoperative pathologic result indicated no detected tumor in the resected mass or the lymph nodes. Thus, the patient was evaluated as having a pathologic complete response (pCR).

**Figure 4:**
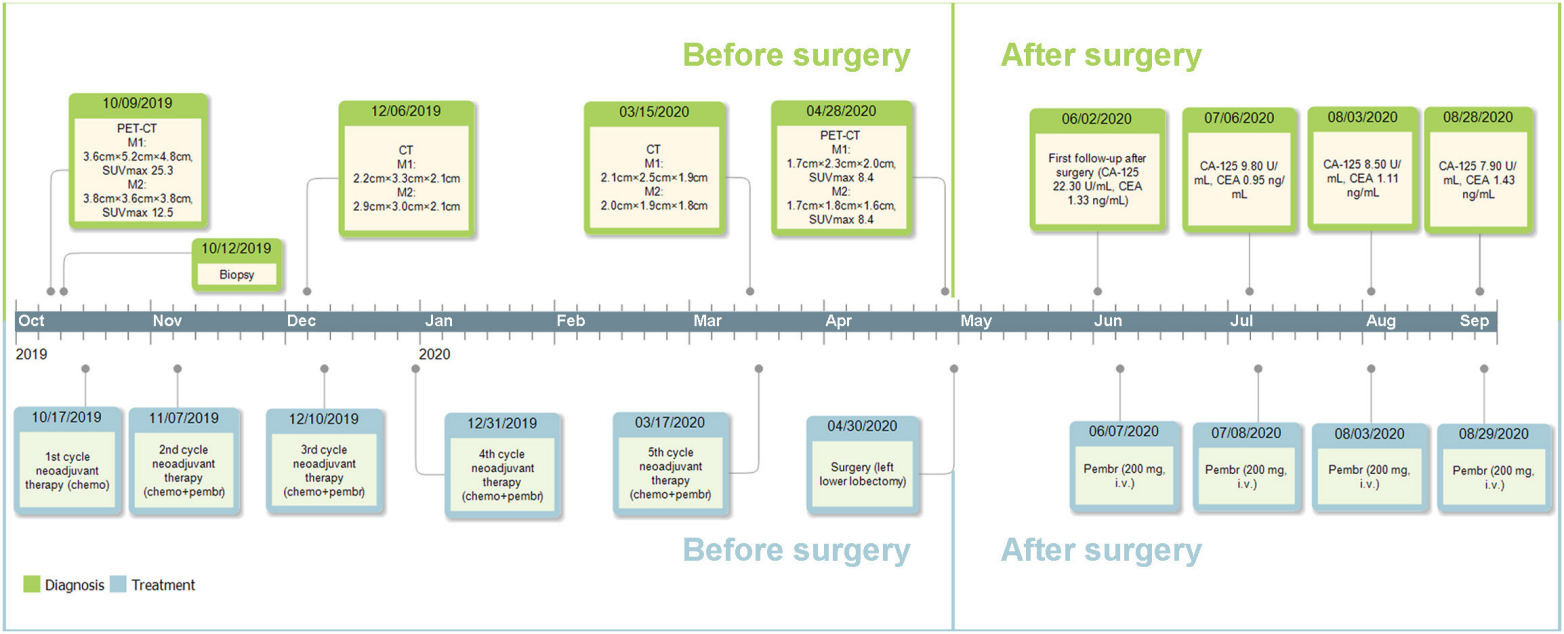
Timeline of the diagnosis and treatment. M1, the mass adjacent to the left hilum; M2, the mass on the basal segment; chemo, chemotherapy; pembr, pembrolizumab; CA-125, carbohydrate antigen 125; CEA, carcino-embryonic antigen; i.v., intravenously.

Postoperative follow-up of the patient was done on June 2nd, 2020, with no recurrent or metastatic signs on chest CT, cranial CT, abdominal ultrasonography, or urinary system ultrasonography. The patient refused postoperative chemotherapy for fear of its adverse effects and received intravenous injection of 200 mg pembrolizumab on June 7th, July 8th, August 3rd, and August 29th, 2020; reported adverse effects included mild cough, vomiting, and decreased platelet count (103.00 × 10^9^/L on August 4st, 2020 and 83.00 × 10^9^/L on August 29st, 2020), which were all alleviated automatically or after symptomatic treatment. Carbohydrate antigen 125 and carcino-embryonic antigen were 22.30 U/mL and 1.33 ng/mL on June 5th, 9.80 U/mL, 0.95 ng/mL on July 6th, 8.50 U/mL, 1.11 ng/mL on August 3rd and 7.90 U/mL, 1.43 ng/mL on August 28th, respectively. Further data were not available. Major events of this case were summarized in Figure 4.

## Discussion

Preoperative treatment could be of potential disadvantage to patients with resectable NSCLC, but it could also have some benefits for these patients. Meanwhile, it might also delay surgery and make the tumor unresectable (9). There are some studies showing that preoperative chemotherapy plus PD-1 inhibitor could benefit patients with resectable NSCLC, despite the lack of complete data. Preoperative nivolumab plus chemotherapy has resulted in an MPR rate of 46.2% (6/13) and a pCR rate of 38.5% (5/13), respectively; the overall response rate was 46.2% (6/13) (10). In contrast, the overall response rate and pCR rate for neoadjuvant pembrolizumab plus chemoradiation were 75.0% (6/8) and 66.7% (4/6), respectively (7). Thus, the combination of anti-PD-1 antibody and chemotherapy as a neoadjuvant therapy is a reasonable plan.

Pembrolizumab has previously been reported to lead to pCR in NSCLC. In one case, neoadjuvant pembrolizumab administration resulted in pCR in a patient with late stage lung adenocarcinoma (11). In another case, adding pembrolizumab to neoadjuvant chemotherapy also yielded a pCR result in a stage IIIA NSCLC patient (12). In this case, combination of pembrolizumab and neoadjuvant chemotherapy resulted in pCR. The satisfying effect of the anti-PD-1 antibody might be attributed to the relatively high PD-L1 expressions in tumor cells in these patients, with TPS being 41.78, 100, and 80%, respectively. However, the special part of this case lies not only in the pCR of tumor, but also in the avoidance of pneumonectomy. The response of the tumor changed the operation plan from pneumonectomy to lobectomy and avoided resection of the left lung. Considering the relatively older age of this patient, the change of the surgery plan is of practical significance to the maintenance of his life quality in the future.

The disadvantage of this report lies in the lack of control, so that the effect of the combinatorial preoperative treatment could not be compared to that of neoadjuvant chemotherapy or pembrolizumab alone. In Keynote-189, pembrolizumab plus chemotherapy as a first-line therapy has shown better median PFS (9.0 months), median overall survival (22.0 months), overall response rate (48.0%, 197/410), and disease control rate (84.6%, 347/410) compared to those of patients receiving placebo plus chemotherapy (4.9 months; 10.7 months; 19.4%, 40/266; 70.4%, 145/206). However, there has been no clinical study comparing the effect of anti-PD-1 antibody plus chemotherapy as a neoadjuvant therapy to that of chemotherapy alone on NSCLC yet. Considering the satisfying anti-tumor activity of neoadjuvant anti-PD-1 antibody in NSCLC in various studies, the effect might be attributed to pembrolizumab administration to a large extent (13, 14).

The addition of pembrolizumab to neoadjuvant chemotherapy has shown promising anti-tumor effect on triple-negative breast cancer (15). Thus, the addition of pembrolizumab to neoadjuvant chemotherapy in NSCLC might also be a satisfactory choice in clinical practice. Future clinical trials with large sample sizes are worth exploring. In conclusion, the case has provided a potential satisfying neoadjuvant plan for NSCLC in clinical practice.

## Data Availability Statement

The raw data supporting the conclusions of this article will be made available by the authors, without undue reservation.

## Ethics Statement

Written informed consent was obtained from the patient for publication of this case report and any accompanying images.

## Author Contributions

LZ and WJ: conceptualization and study design. LZ and WM: data collection. WM: literature research. LZ: manuscript drafting. WJ and QG: revision. QG: funding. All authors contributed to the article and approved the submitted version.

## Conflict of Interest

The authors declare that the research was conducted in the absence of any commercial or financial relationships that could be construed as a potential conflict of interest.
